# Direct Thermal Method to Characterize the Material Efficiency of Electrocaloric Lead Scandium Tantalate Multilayer Ceramic Capacitors

**DOI:** 10.3390/ma18091924

**Published:** 2025-04-24

**Authors:** Sabrina Unmüßig, David Bach, Julius Metzdorf, Patrick Corhan, Sakyo Hirose, Kilian Bartholomé

**Affiliations:** 1 Fraunhofer Institute for Physical Measurement Techniques IPM, Georges-Köhler-Allee 301, 79110 Freiburg, Germany; sabrina.unmuessig@ipm.fraunhofer.de (S.U.); david.bach@ipm.fraunhofer.de (D.B.); julius.metzdorf@ipm.fraunhofer.de (J.M.); patrick.corhan@ipm.fraunhofer.de (P.C.); 2 Murata Manufacturing Co., Ltd., Kyoto 617-0833, Japan; h_sakyo@murata.com

**Keywords:** electrocaloric, thermal characterization, FOM, efficiency, caloric materials, PST, caloric cooling

## Abstract

In this study, we characterize electrocaloric lead scandium tantalate (PST) samples by means of the adiabatic temperature change $\Delta T_{\mathrm{ad}}$ and the dissipative heat $q_{\mathrm{diss}}$ with a direct thermal method. The figure of merit (FOM), defined as the ratio between the adiabatic temperature change and the thermal hysteresis, quantifies the losses of the material. Additionally, it is also possible to draw conclusions on the efficiency of a caloric cooling system based on the regenerator or cascaded approach. The maximum adiabatic temperature change of the measured samples results in $\Delta T_{\mathrm{ad},\max}=(1.39\pm 0.02)$ K and the dissipative heat yields $q_{\mathrm{diss}}=(0.39\pm 0.05)\;J/(kg\;K)$, resulting in an FOM=(5.1±0.2). The efficiency for an ideal cascaded system is given by $\eta_{\mathrm{cas}}=0.56$, and for the ideal regenerator, the efficiency is given by $\eta_{\mathrm{reg}}=0.84$. The results demonstrate that the PST material in this study exceeds the maximum FOM in the literature by 34%.

## 1. Introduction

Electro-, elasto- and magnetocaloric cooling systems are a promising alternative to standard compression-based approaches [1,2,3,4,5,6,7,8,9]. While the overall efficiency of superior compression-based systems is approx. 50% of the Carnot efficiency [10], prototypes of caloric cooling systems have achieved efficiencies over 60% in the last years, as demonstrated by Chaudron et al. for a magnetocaloric cooling system [11] and by Li et al. for an electrocaloric cooling system [12]. Caloric cooling systems make use of the so-called caloric effect [13,14,15], in which a first- or second-order reversible phase transition induces a temperature change in the caloric material. In the case of electrocaloric materials, the effect occurs by applying or removing an electric field, which induces a phase transformation from the ferroelectric to the paraelectric phase or vice versa [16]. Electrocaloric heat pumps or cooling systems such as by Metzdorf et al. [17], Li et al. [12], or Ma et al. [18,19] use the caloric effect by cycling the electric field and pumping the heat the material generates. For the commercialization of caloric heat pump technology, it is imperative that the system efficiencies are equivalent to or exceed those of compressor-based systems. In this context, the utilization of highly efficient caloric materials that demonstrate minimal dissipative heating during operation, alongside highly efficient power electronics, is particularly critical. While Moench et al. [20] have demonstrated the feasibility of developing highly efficient electrocaloric power electronics with efficiencies reaching up to 99.74%, the efficiency of the caloric materials themselves may still pose a limiting factor [20,21]. Even very small amounts of dissipative heat $q_{\mathrm{diss}}$, as demonstrated by Hess et al. [22] and Masche et al. [23], can have a significant impact on the overall system efficiency. The amount of dissipative heat of an electrocaloric material depends, for example, on factors such as the field strength and direction [24], as well as the manufacturing process [25,26]. Therefore, having a reliable and time-effective measurement technique available to characterize the dissipative heat and efficiency of electrocaloric materials is of great importance, especially with regard to material optimization studies. To determine the efficiency of the caloric material, this paper uses a figure of merit (FOM) introduced by Hess et al. [22] and Schipper et al. [21], which relates the thermal losses of the material to the adiabatic temperature change. With the help of the FOM, the maximum system efficiency using a certain material can be determined for both ideal regenerator and cascaded systems (see, e.g., ref. [27] for differentiation). A higher FOM is associated with a higher efficiency. However, to correctly ascertain the FOM, it is essential to accurately determine its characterization parameters. While the adiabatic temperature change can be measured directly using a thermocouple (TC), the dissipative heat losses of the material can be determined using a method developed by Schipper et al. [28] for magnetocaloric materials. This approach is based on the effect of self-heating of caloric materials under field cycling due to irreversible temperature changes. The observed increase in temperature is partially attributed to heating caused by hysteresis losses of the material and, in certain cases, also arises from Joule heating [22,29,30]. In this paper, the method presented by Schipper et al. is adapted for electrocaloric ceramic multilayer capacitors (MLCs) to characterize ${\mathrm{PbSc}}_{0.5}$${\mathrm{Ta}}_{0.5}$$O_{3}$ (PST) MLCs at different electric field strengths and temperatures. The selected PST electrocaloric material represents the state-of-the-art electrocaloric ceramic. It has been utilized in various electrocaloric cooling demonstrators documented in the literature (see, for example, Metzdorf et al. [17]). Similar approaches to determine thermal losses in electrocaloric materials have also been described by Fischer et al. [31], who investigated the single-crystalline relaxor material 0.9Pb(${\mathrm{Mg}}_{1/3}$${\mathrm{Nb}}_{2/3}$)$O_{3}$-0.1${\mathrm{PbTiO}}_{3}$ (PMN-10PT), and Bradeško et al. [29], who analyzed the relaxor Pb(${\mathrm{Mg}}_{1/3}$${\mathrm{Nb}}_{2/3}$)$O_{3}$ (PMN) and two different rhombohedral ferroelectric Pb(Zr,Ti)$O_{3}$ (PZT) compositions.

## 2. Materials and Methods

### 2.1. Lead Scandium Tantalate Samples

**Figure 1:**
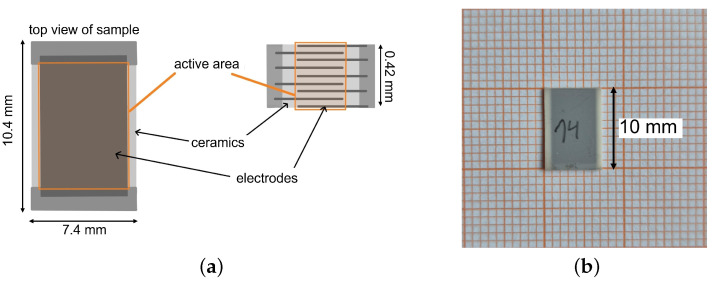
(**a**) Schematic setup of an electrocaloric multilayer capacitor (MLC). The image shows a top and a side view of an MLC. The active area of one of the electrodes is marked orange. The active layer thickness is approx. 38 μm. (**b**) PST sample on graph paper.

The studied components consist of nine single electrodes which are positioned with an offset to each other. This is depicted by the side view of the schematic setup of one component in Figure 1a. An image of one PST sample is shown in Figure 1b. The electrodes are connected to each other via a contact area. The active area of an electrocaloric MLC is the part of the ceramic that is situated between the electrodes and exhibits an electrocaloric effect when an electric field is applied. The inactive area of the MLC refers to the part of the ceramic (and, strictly speaking, the electrodes) that does not show a (significant) electrocaloric effect under the influence of an electric field at the electrodes. This inactive area is primarily heated or cooled by heat conduction from the active to the inactive material, generally comprising edge regions or inactive surface layers. The inactive area serves to enhance the dielectric strength of the multilayer component.

**Figure A1:**
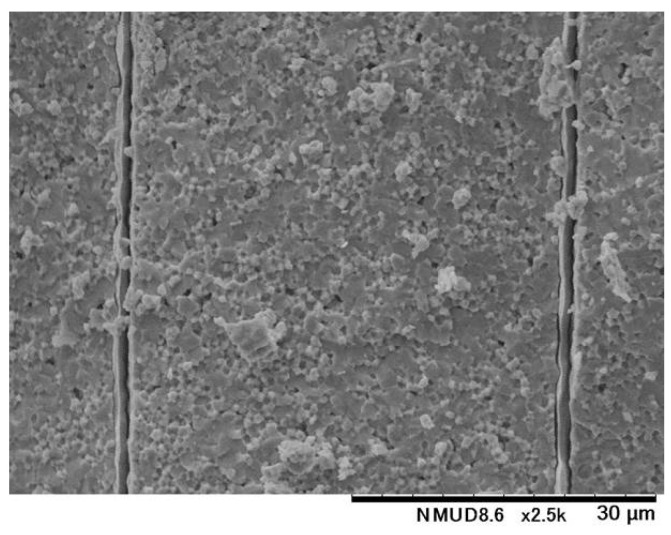
SEM image of a PST sample.

**Figure A2:**
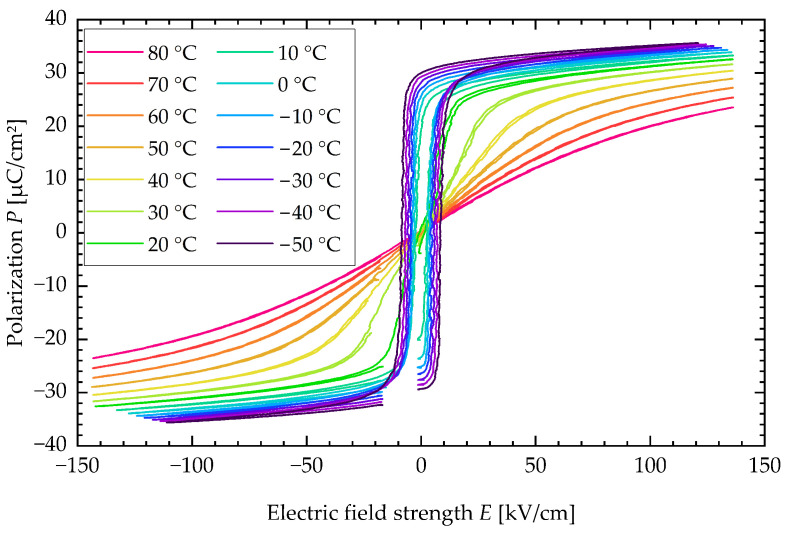
*P*-*E*-loop of PST sample for various sample temperatures.

The materials used in this study are ${\mathrm{PbSc}}_{0.5}$${\mathrm{Ta}}_{0.5}$$O_{3}$ (PST) multilayer capacitor samples fabricated by Murata Manufacturing company. One multilayer capacitor has the dimensions of 10.4 × 7.4 mm with a thickness of approx. 420 μm. The active layer thickness is approx. 38 μm. Nouchokgwe et al. measured an active area (the overlap of the electrodes) of the same components of $48.7\;mm^{2}$ [32]. The ceramic layer thickness results in 38.6 μm, which yields an active volume of $16.9\;mm^{3}$. The density of the material (determined by X-ray diffraction) is $\rho =9080\;kg/m^{3}$ [33]. With the density of the PST material, the active mass of the ceramic layers is 0.15 g. For more information on the PST material, see Appendix A (an SEM image is shown in Figure A1 and the polarization curve is shown in Figure A2). During this study, two different batches of the same material were used. The separate results for the single batches can be found in Appendix A, while the summarized results can be found in the main part of this manuscript. Batch 1 contains three separate samples while batch 2 contains two different samples of the same material. All the samples were characterized at two sample temperatures: 25 and 30 °C. The samples were characterized at four different voltages (U=100 V, 150 V, 200 V and 250 V), leading to characterization measurements at the following field strengths: E=2.6 V/μm, 3.8 V/μm, 5.1 V/μm and 6.4 V/μm.

### 2.2. Measurement Set Up

**Figure 2:**
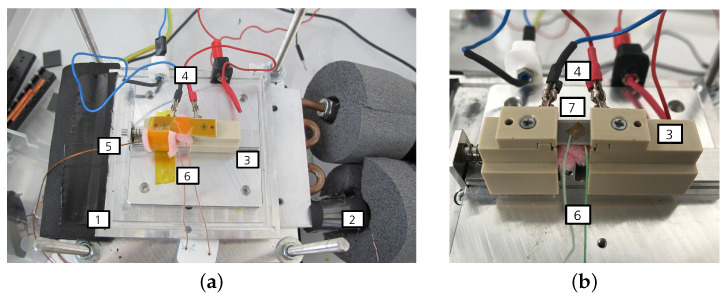
Measurement setup. (**a**) Measurement setup for the characterization of the electrocaloric sample. (**b**) Close-up view of the measurement setup. (1) Heat exchange plate, (2) thermocouple placed on the inflow of the recirculating chiller unit, (3) sample holder, (4) electrical contacts, (5) thermocouple for the surroundings, (6) thermocouple placed on the sample and (7) sample with affixed thermocouple on top.

Figure 2 shows the measurement setup. Thereby, Figure 2b shows a close-up view of Figure 2a.

The electrocaloric components are placed inside of a component chamber made from perspex to avoid touching the active and under-voltage parts of the measurement setup. On the side, there are openings for TCs (Omega, Deckenpfronn, Germany, type K) and for the tubes of a recirculating chiller unit (F32-MC, Julabo, Seelbach, Germany) which regulates the temperature inside of the chamber. The sample holder (3) is positioned atop a heat exchange plate (1), which is connected to the tubing of the chiller unit (2). Within the sample holder, two copper sheets are integrated and connected to the electrical contacts of the component. The component itself is secured using springs to ensure stable positioning. To measure the temperature of the component accurately, a TC is affixed to the top of the component, utilizing a Kapton film to enhance thermal contact and ensure precise temperature measurements. Kapton film is electronically insulating and has a low specific thermal conductivity. The TC has a diameter of 0.05 mm granting the advantage that the heat flow from the TC is small and the response time is short [34]. There is also a TC on the sample holder to measure the temperature of the surroundings $T_{a}$ (5). The diamater of this TC is 0.25 mm since the fluctuations are irrelevant in this case because only the mean temperatures matter. Lastly, the temperature on the chiller tubes (2) is measured as well. The TCs are connected to the data logger (DAS 801, Sefram, Saint-Étienne, France) via a plug connection and an extension cable. The voltage signal that arises due to the thermolectric voltage is converted into a temperature internally. For a better thermal stabilization, the chiller tubes are wrapped with insulation material. The component and the TCs are encased with foam material so that as little heat as possible is transferred to the surroundings. To protect the sample from forced convections, another small perpex cover is placed on top of the sample holder (not depicted in the figure to maintain clarity of the setup). The voltage supply for measurements is the SM1500-cp-30 (Delta Elektronika, Zierikzee, The Netherlands).

### 2.3. Characterization Measurements and Error Propagation

#### 2.3.1. Adiabatic Temperature Change

**Figure 3:**
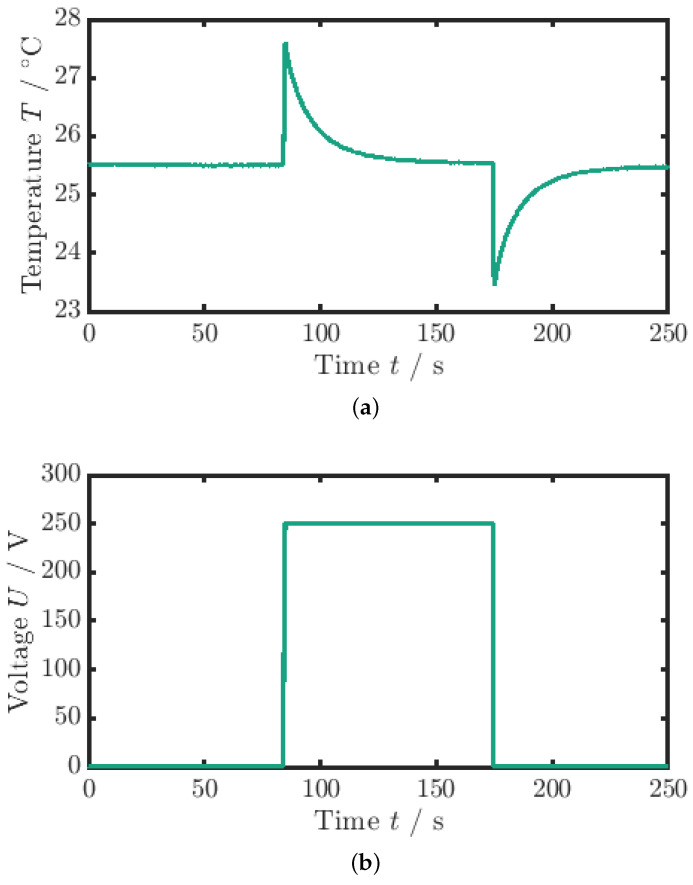
Measurement data for the adiabatic temperature change. (**a**) Temperature profile. (**b**) Voltage profile (the maximum voltage is 250 V). The adiabatic temperature change is the temperature difference between the minimum peak and the mean value of the surrounding temperature.

For the measurement of the adiabatic temperature change ($\Delta T_{\mathrm{ad}}$), a quasi-instantaneous voltage signal is applied, as can be seen in Figure 3b. The quasi-instantaneous voltage jump (t=200 ms) causes the material to heat up from the ambient temperature by the adiabatic temperature change (see Figure 3a).

The electric field, or voltage, is maintained constant until the MLC temperature has equilibrated with the ambient temperature again (t=90 s). If the process was completely adiabatic, the temperature would remain constant. Hence, due to the coupling of the sample to the environment and the response time of the TC, the measured $\Delta T_{\mathrm{ad}}$ values are slightly underestimated. Subsequently, the voltage, and thus the electric field, is removed, and the temperature decreases by the adiabatic temperature change below the ambient temperature. After that, a second waiting period is applied until the sample temperature again equilibrates with the ambient temperature. The evaluation of the adiabatic temperature change measurement is based on the average of the field removal curves obtained from three consecutive measurements for each sample. Specifically, for each measurement, the maximum temperature change during the cooling phase is evaluated, and the average of these temperature changes is calculated to determine a representative adiabatic temperature change for the sample. This process ensures that variability between measurements is minimized, providing a more accurate estimate of the adiabatic temperature change. Therefore, the adiabatic temperature change is calculated via

(1) $$\Delta T_{\mathrm{ad},\mathrm{off}}\;=\;T_{\min}-T_{a},$$

where $T_{\min}$ is the mean value of the minimum temperature and $T_{a}$ is the mean value of the ambient temperature.

#### 2.3.2. Dissipative Heat

The specific dissipative heat loss $q_{\mathrm{diss}}$ is determined according to the method published by Schipper et al. [28] for magnetocaloric materials. However, some modifications were made to the measurement procedure and data analysis to accommodate the characterization of electrocaloric MLCs. According to Hess et al. [22] and Schipper et al. [28], in a single cycle, the temperature of the caloric material rises by the irreversible temperature change $\Delta T_{\mathrm{irr}}$. When the material is being cycled continuously, the temperature will exponentially approach a steady state at a higher temperature. At this steady-state temperature, the heat flux ${\dot{Q}}_{\mathrm{out}}$ out of the sample into the colder environment increases to the same value as ${\dot{Q}}_{\mathrm{diss}}$, which is the dissipative heating during cycling. In case there is no additional heat flow caused by Joule heating ${\dot{Q}}_{\mathrm{Joule}}$, ${\dot{Q}}_{\mathrm{diss}}$ is equal to ${\dot{Q}}_{\mathrm{hyst}}$, which denotes the hysteresis loss heating of the material (${\dot{Q}}_{\mathrm{diss}}$ = ${\dot{Q}}_{\mathrm{hyst}}$ + ${\dot{Q}}_{\mathrm{Joule}}$) (see [22,28,35,36] for further information). The underlying heat flow balance accounts for all the relevant heat flows in the system:

(2) $$\sum \dot{Q}\;=\;{\dot{Q}}_{\mathrm{diss}}-{\dot{Q}}_{\mathrm{cal}}-{\dot{Q}}_{\mathrm{out}},$$

in which ${\dot{Q}}_{\mathrm{cal}}$ is the heat flux going into the caloric material. From this heat balance, the following differential equation can be derived:

(3) $$m\;\cdot \;c\;\cdot \;\frac{dT}{dt}\;=\;q_{\mathrm{diss}}\;\cdot \;m\;\cdot \;f-\left(T-T_{a}\right)\;\cdot \;K_{\mathrm{th}}.$$

${\dot{Q}}_{\mathrm{diss}}$ is determined by the specific dissipative heat energy $q_{\mathrm{diss}}$ ($q_{\mathrm{diss}}=\Delta T_{\mathrm{irr}}\;\cdot \;c$, where *c* is the specific heat capacity), the mass of the caloric material *m* and the frequency *f* by which the material is being cycled. The heat flux out of the material is given by the thermal conductance between the caloric material and environment $K_{\mathrm{th}}$ and the ambient temperature $T_{a}$. Solving this equation, we obtain

(4) $$T\left(t\right)\;=\;\frac{q_{\mathrm{diss}}\;\cdot \;f}{\kappa \;\cdot \;c}+T_{a}-\left(\frac{q_{\mathrm{diss}}\;\cdot \;f}{\kappa \;\cdot \;c}+T_{a}-T_{0}\right)\;e^{-\kappa \;\cdot \;t},$$

where $T_{0}$ is the starting temperature, *c* is the specific heat capacity and the coupling constant κ is given by

(5) $$\kappa \;=\;\frac{K_{\mathrm{th}}}{c\;\cdot \;m}\;,$$

Thus, by experimentally quantifying the coupling constant κ and then measuring the time dependence of the temperature of the caloric material while applying a sinusoidal field change with frequency *f*, the specific dissipative heat energy $q_{\mathrm{diss}}$ and thereby the FOM (Equation (11)) and the efficiency of the caloric material (Equation (12)) can be determined.

##### Dissipative Heat: Measurement Process

**Figure 4:**
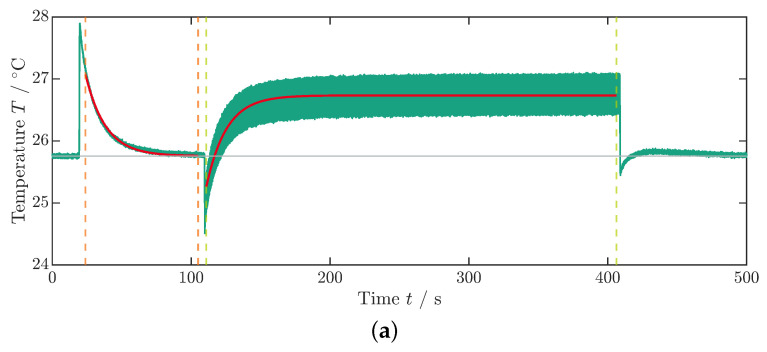

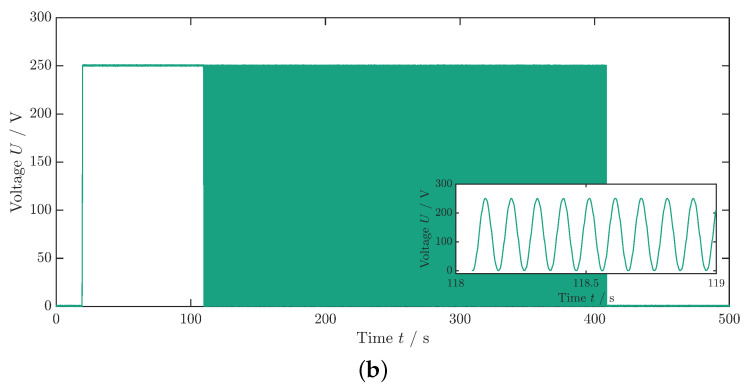
Measurement data for the dissipative heat measurement. (**a**) Temperature profile of the dissipative heat measurement. The orange dashed lines represent the time steps $t_{1}$ and $t_{2}$, whereas the green dashed lines represent the time steps $t_{3}$ and $t_{4}$. The curve fit is illustrated by the red lines. The data that are depicted in this graph are already corrected with the surrounding temperature, as described in Appendix A. (**b**) Voltage profile of the dissipative heat measurement. The inset shows the periodic change in the voltage signal. In this example, the maximum voltage is 250 V.

As previously described in the measurement of $\Delta T_{\mathrm{ad}}$, the sample is initially subjected to a constant electric field for t=90 s using a step function with a rise and fall time of t=200 ms (Figure 4b). Subsequently, a transition occurs to a cyclic voltage application: the sinusoidal profile (see inset Figure 4b) of the voltage set to f=10 Hz, and thus the electric field is maintained for approximately t=300 s until the average component temperature no longer rises (Figure 4a). In the equilibrium state, the averaged temperature takes a value that is greater than that of the environment or ambient temperature $T_{a}$ (t→∞). Subsequently, the electric field is switched off, leading to a sudden decrease in temperature followed by a second temperature equalization process to the ambient temperature.

##### Dissipative Heat: Data Evaluation

The ambient temperature $T_{a}$ is averaged from the temperature profile (see Figure 4a) up to the time when the voltage step is implemented (Figure 4b). To determine the thermal coupling constant to the environment κ, the exponential temperature drop under a constant field over time *t* is fitted to an exponential decay function:

(6) $$T\left(t\right)\;=\;T_{a}-\left(T_{a}-T_{0}\right)\;\cdot \;e^{-\kappa \;\cdot \;\left(t-t_{0}\right)},$$

Here, $t_{0}$ and $T_{0}$ denote the starting time and temperature, respectively. For the fit boundaries, the time frame between the two orange dashed lines at $t_{1}$ and $t_{2}$ in Figure 4a is used. Differing from Schipper et al. [28], in this paper, the first cooling phase is taken for fitting since the cycling period was ended with a field-off setting. Additionally, in order to mitigate an influence of inhomogeneous temperatures in the MLC on κ, the left boundary of the fit at $t_{1}$ is set with a time offset relative to the temperature peak to allow for a temperature equalization process of the active and inactive parts of the MLC (see Figure 1a). The curve fit is illustrated in Figure 4a by the red lines. Since, under the tested conditions, PST showed no visible Joule heating (see Figure 3a and Figure 4a), a Joule heating term was omitted in the fit function of the exponential temperature decay (see [35,36] for further details).

**Figure A3:**
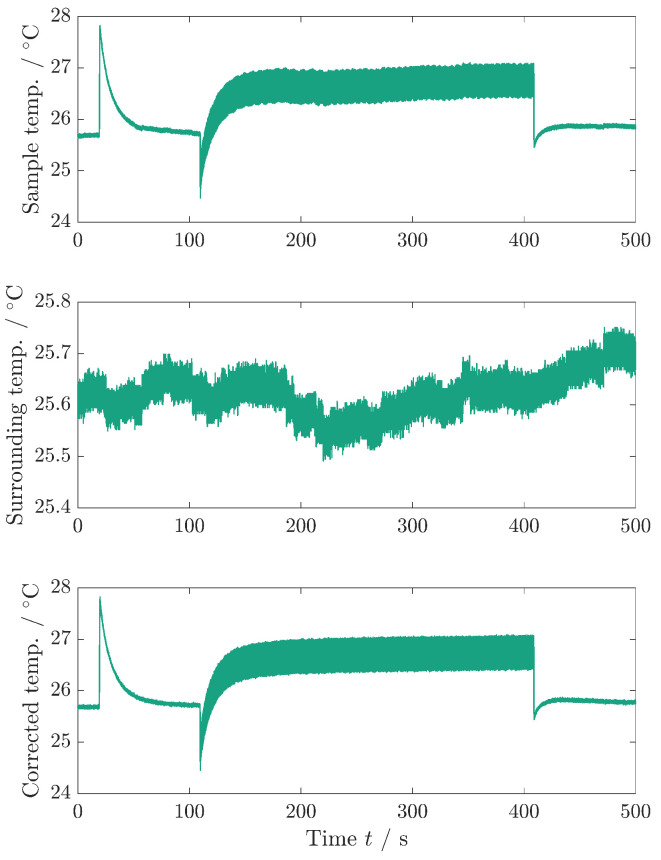
Temperature correction of the sample temperature. **Upper graph**: sample temperature as recorded. **Middle graph**: surrounding temperature, measured with a different TC. **Bottom graph**: sample temperature that is corrected with the surrounding temperature. Since both TCs have the same reference junction compensation in the data logger, the noise of the sample temperature can be reduced by correcting the sample temperature with the surrounding temperature. The bottom graph is used for fitting the model equations to the data and is the same data as depicted in Figure 4a.

Similarly, the temperature development under cycling starting at $t_{3}$ is fitted using Equation (4). In this case, the coupling constant κ is passed as a fixed value. The dissipative heat loss $q_{\mathrm{diss}}$ is determined as a free parameter from the fit (boundaries indicated by green dashed lines between $t_{3}$ and $t_{4}$ in Figure 4a). The right boundary of the fit is determined just before the temperature drop that occurs when the voltage is turned off at the end of the measurement procedure (Figure 4a,b). To enhance the measurement accuracy and mitigate the effects of internal fluctuations within the data logger on signal quality, the following procedures were implemented:

1. The noise present in the sample temperature signal was attenuated by subtracting the ambient temperature from the sample temperature (refer to Figure A3 in the Appendix B).
2. To achieve the correct absolute temperature, the mean ambient temperature, measured prior to the application of the voltage step, was reintroduced to the denoised sample temperature.

This procedure is possible since both TCs have the same reference junction compensation in the data logger. All TC connections were additionally insulated with foam.

#### 2.3.3. DSC Measurement

The specific heat capacity c(T) can be derived from differential scanning calorimetry (DSC) measurements, which provide the heat flow as a function of temperature. The specific heat capacity for the samples characterized in this research was reported by Nouchokgwe et al. [32] as c=310 J/(kg K) at room temperature.

#### 2.3.4. Uncertainty Propagation

The results denoted in the Section 3 are the mean values of single data points. As described in the Section 2, two batches of PST material with a total of 5 samples were characterized during this study. For both the adiabatic temperature change measurements and the dissipative heat measurements as well, the number of measurements conducted per sample, temperature and field strength is typically n=3. Added up, this leaves between N=12 and N=15 measurements per field strength and temperature. The mean value $\bar{x}$ is calculated with

(7) $$\bar{x}\;=\;\frac{1}{N}\sum_{i}x_{i},$$

in which *N* is the number of measurements and $x_{i}$ are the single data points. In our study, *x* is the adiabatic temperature change, the dissipative heat or the FOM. The standard deviation $s_{x}$ is calculated via

(8) $$s_{x}=\sqrt{\frac{1}{N-1}\sum_{i}{\left(x_{i}-\bar{x}\right)}^{2}}.$$

This results in a standard deviation from the mean of

(9) $${\bar{s}}_{x}\;=\;\frac{s_{x}}{\sqrt{N}}.$$

${\bar{s}}_{x}$ is the total statistical uncertainty for one data point. The systematic uncertainty of the adiabatic temperature change is assumed to be $\delta_{\Delta T_{\mathrm{ad}}}=0.1\;K$. The accuracy of the TC can be neglected in this case since the temperature change is the difference between two temperatures measured with the same TC. The systematic uncertainty of the dissipative heat measurement is assumed to be 10% of the measurement value $\Delta_{q_{\mathrm{diss}}}=0.1q_{\mathrm{diss}}$. In principle, one could use the uncertainties of the exponential fit to calculate a systematic uncertainty, but since the total number of data points for this measurement is huge and the model fits the data well, the 95% confidence interval of the fit is in the per-thousand range. Nevertheless, the uncertainty given here is a conservative error estimation. The total error of one data point is the sum of both statistical and systematical uncertainty:

(10) $$s_{\mathrm{total},x}\;=\;s_{x}+\delta_{x}$$

### 2.4. Calculation of the FOM & Efficiency

As described by Hess et al. [22] and Schipper et al. [21], the FOM is calculated via

(11) $$FOM\;=\;\frac{\Delta T_{\mathrm{ad}}}{\Delta T_{\mathrm{hyst}}}=\frac{\Delta T_{\mathrm{ad}}\Delta s_{\mathrm{iso}}}{q_{\mathrm{diss}}}=\frac{\Delta T_{\mathrm{ad}}^{2}c}{q_{\mathrm{diss}}T}.$$

Here, $\Delta T_{\mathrm{hyst}}$ represents the thermal hysteresis, $\Delta s_{\mathrm{iso}}$ denotes the isothermal entropy change and *T* denotes the sample temperature in Kelvin. Since Joule heating can be neglected for PST, the specific hysteresis loss heat energy $q_{\mathrm{hyst}}$ and the specific dissipative heat loss energy $q_{\mathrm{diss}}$ are equal and interchangeable. The ideal efficiency η for a caloric system is described with [21,22]:

(12) $$\eta \;=\;\frac{1}{1+\frac{1}{\alpha FOM}},$$

in which α=1 leads to the efficiency of an ideal regenerator $\eta_{\mathrm{reg}}$ and α=1/4 yields the equation for an ideal cascaded system with an efficiency of $\eta_{\mathrm{cas}}$. The uncertainty of the FOM is calculated via Gaussian error propagation. Since the efficiency results only from a factor of the FOM, we assume the efficiency itself to be without error.

## 3. Results and Discussion

**Figure 5:**
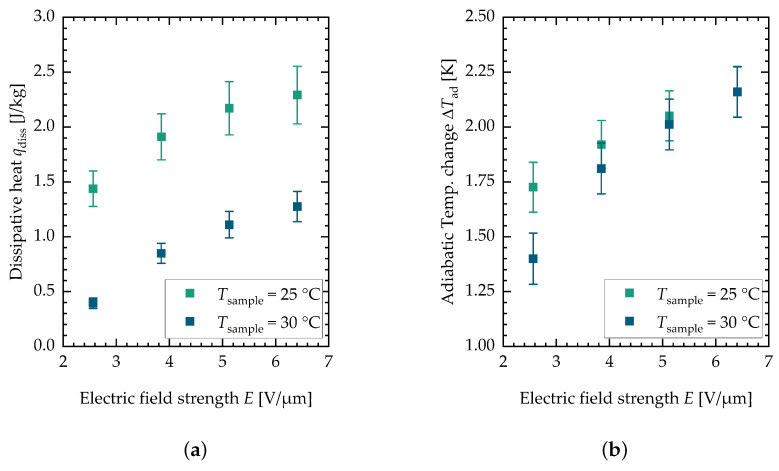
(**a**) Measurement results for the dissipative heat measurement. (**b**) Measurement results for the adiabatic temperature change measurement. Each data point is the mean value of minimal twelve and maximal fifteen single measurements. The error bars are the sum of the systematic and the statistical error.

Figure 5 shows the results for the dissipative heat measurements (see Figure 5a) and the adiabatic temperature change measurements (see Figure 5b).

**Figure 6:**
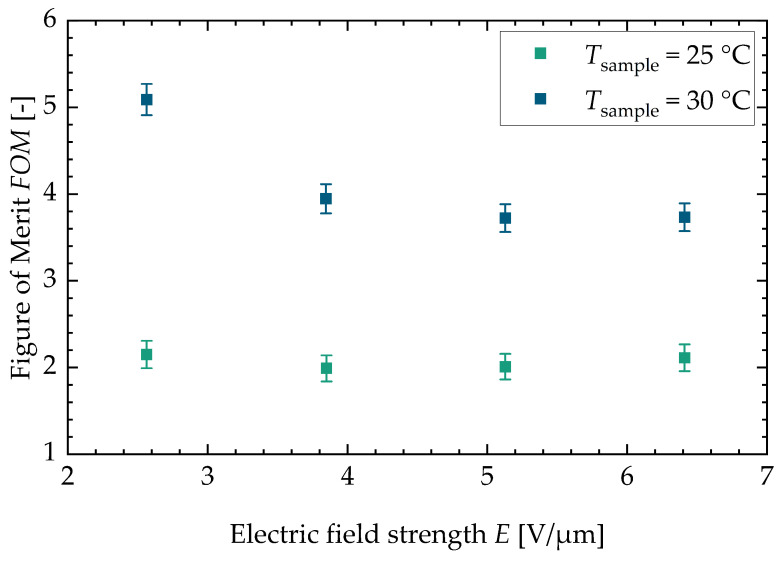
Figure of merit for both sample temperatures versus the electric field strength. Both sample temperatures show a decrease in the figure of merit for higher electric field strengths. The maximum value can be found at a sample temperature of $T_{\mathrm{sample}}=30\;{}^{\circ}C$ and is FOM=(5.1±0.2).

These values are plotted against the electric field strength for two different sample temperatures, namely $T_{\mathrm{sample}}=25\;{}^{\circ}C$ and $T_{\mathrm{sample}}=30\;{}^{\circ}C$. The data points depicted are mean values of minimal twelve and maximal fifteen single measurements. The data from the two different batches can be found in Appendix A in Figure 4b for the adiabatic temperature change and in Figure 4a for the dissipative heat. It can be seen that the adiabatic temperature change increases for both sample temperatures with the electric field strength. For lower field strengths, the value is higher for lower sample temperatures. The dissipative heat measurements show a different behavior. For the lower sample temperature, the dissipative heat is bigger in general. This is consistent with measurements of the isothermal electrical polarization of PST performed by Nair et al. [37], where the hysteresis but also the maximum polarization decreased with an increase in temperature. The measurement data indicate that the phase transition is incomplete at the examined field strengths and sample temperatures. This can be observed in both the adiabatic temperature change and dissipative heat measurements. However, this study only explored a limited range of field strengths and sample temperatures in order to minimize damage to the samples (similar electric field strengths, for this reason, were also used in the active electrocaloric heatpipe system developed by Metzdorf et al. [17]). Both the dissipative heat and the adiabatic temperature change eventually reach a plateau at higher field strengths, indicating that the phase transition is completed (see the flattening of the curve above the 6 V/μm measurements). The observed decrease in both the dissipative heat and adiabatic temperature change at higher sample temperatures is attributed to the fact that the phase transition requires higher electric field strengths. Consequently, this results in a shift in the curves along the *x*-axis toward these field strengths. Further investigation at additional sample temperatures would be beneficial to achieve a clearer representation of the data. However, PST has the characteristic of exhibiting a relatively narrow transition peak at low electric field strengths. The application of caloric materials for cooling purposes is typically centered around room temperature, which is the reason for selecting these specific sample temperatures. For multilayer components, it could be observed that they behave more like a relaxor exhibiting a broader temperature range of the caloric effect when the field strength is increased to ranges of E=15 V/μm to 30 V/μm [37]. However, the peak of the caloric effect in consequence shifts more toward higher temperatures. Figure 6 shows the FOM against the electric field strength for both sample temperatures.

**Figure 7:**
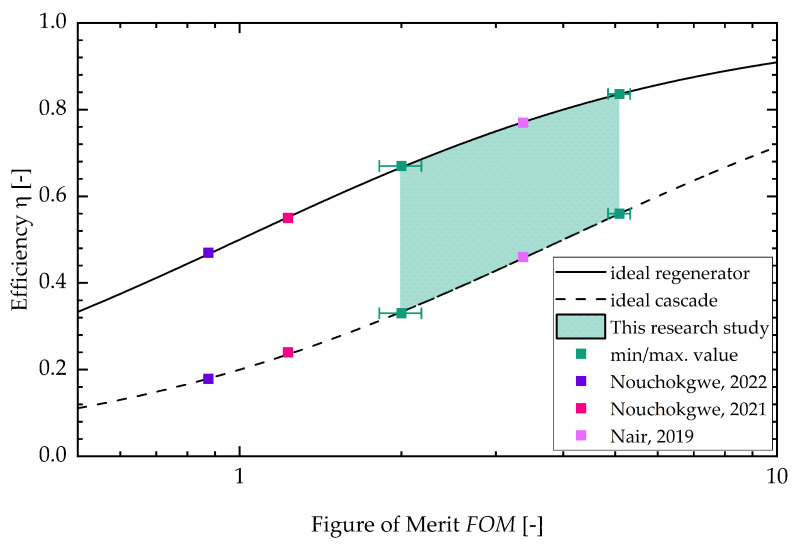
Efficiency versus FOM. The literature values (Nouchokgwe et al. (violet data points) [32], Nouchokgwe et al. (pink data points) [33] and Nair et al. [37]) were calculated via thermal hysteresis and the adiabatic temperature change. The green interval depicts the result interval from the measurements, as demonstrated in this study, and the minimum and maximum value for the FOM is depicted by data points.

**Figure A5:**
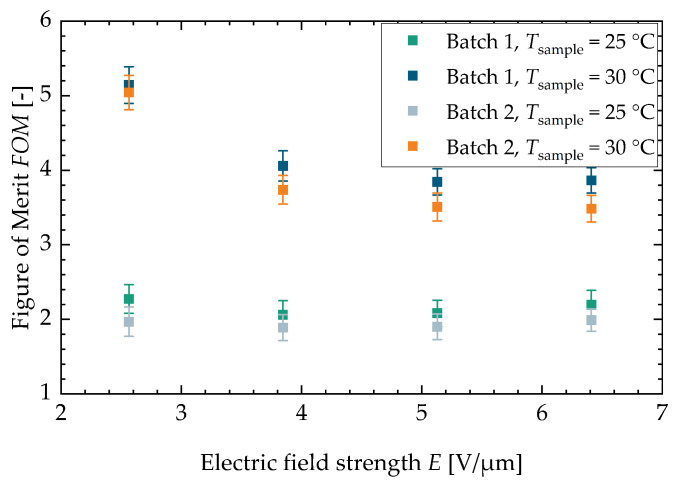
FOM drawn against the electric field strength for both characterized batches.

In Appendix C, the figure for the two different batches can be found (see Figure A5). With a higher sample temperature, the figure of merit increases. The FOM stays constant for the lower sample temperature with increasing field strength. For the higher sample temperature, the FOM decreases with increasing field strength. This is due to the fact that the dissipative heat increases faster with increasing field strength than the square of the adiabatic temperature change. Lastly, Figure 7 shows the efficiency of an ideal cascaded system resp. a regenerator system drawn against the FOM. The literature values calculated from three research studies about electrocaloric PST material are shown as well (Nouchokgwe et al. (violet data points) [32], Nouchokgwe et al. (pink data points) [33] and Nair et al. [37]). The maximum and minimum value for the FOM of this study is depicted by green data points. The green area denotes that the rest of the measurements lie in this interval. The maximum FOM from this study is $FOM_{\max}=\left(5.1\pm 0.2\right)$, leading to an ideal cascade efficiency of $\eta_{\mathrm{cas}}=56$% and an ideal regenerator efficiency of $\eta_{\mathrm{reg}}=84$%. This is in agreement with the experimentally determined system efficiency published by Li et al. [12] for a PST regenerator system. The maximum FOM from this study exceeds the maximum FOM reported from the literature, $FOM_{\max,\mathrm{lit}}=3.37$ [37], by 34%. The FOM for the literature values (as can be seen in the study by Schipper et al. [21]) was calculated via the thermal hysteresis and therefore with DSC measurements and with the adiabatic temperature change. This is different in comparison to our approach in which the FOM is calculated with the dissipative heat.

## 4. Conclusions

In this study, we adapted a material characterization methodology from the field of magnetocalorics for use in electrocalorics. We examined electrocaloric PST multilayer components (MLCs) at two different sample temperatures and for several electric field strengths with regard to the dissipative heat and the adiabatic temperature change. The adiabatic temperature change was measured directly with a thermocouple whereas a self-heating approach was used to determine the dissipative heat. Along with the sample temperature and the specific heat capacity at that temperature, we calculated a figure of merit. Additionally, we determined the ideal efficiency for both a regenerator and a cascaded approach within an electrocaloric cooling system. This study indicates that the FOM has a maximum at a field strength of E=2.6 V/μm and a sample temperature of $T_{\mathrm{sample}}=30\;{}^{\circ}C$ and results in $FOM_{\max}=5.1\pm 0.2$. This is 34% larger than the maximum FOM for PST as found in the literature. Moreover, the findings demonstrate that there is already the potential to build highly efficient systems with the currently available electrocaloric material PST at certain temperature ranges and electric field strengths. Without any additional losses, a system equipped with PST MLCs could reach maximum efficiencies (relative to the Carnot efficiency) in the range of up to about 50 to 80% depending on the system configuration and design. The described measurement method is characterized by a high level of accuracy (a FOM measurement error of 4%) and simplicity and thus enables different (electro)caloric materials to be compared with each other. This is of particular importance in the process of material optimization to further improve their performance and efficiency. Nevertheless, the method offers the advantage of also being able to precisely determine the dissipative heat loss of materials that have a very low field hysteresis. This can make it difficult to determine the values from polarization-electric field measurements. It is possible to investigate any type of caloric material regarding its dissipative heat using the self-heating approach. Accordingly, further electrocaloric materials can also be studied with this method.

Since, apart from temperature, the behavior of PST MLCs largely depends on the applied electric field strength, further measurements could additionally focus on characterization at higher field strengths above E=10 V/μm up to E=30 V/μm, where the caloric effect has a broader temperature range. 

## Figures and Tables

**Figure A4:**
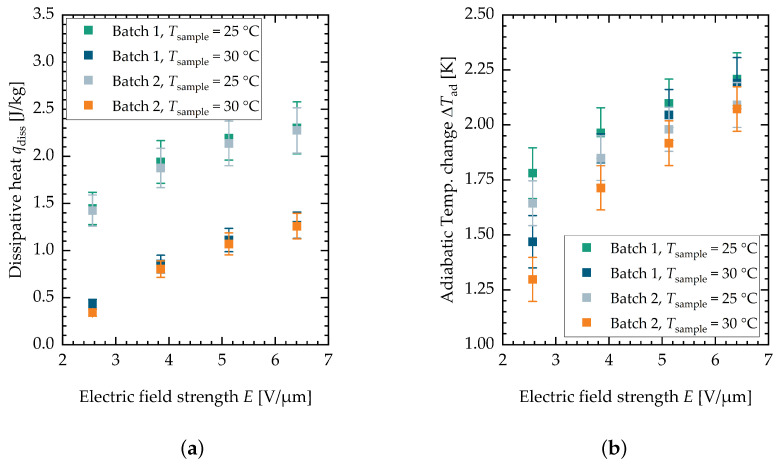
(**a**) Summary of the dissipative heat measurement. (**b**) Summary of the adiabatic temperature change measurement. Both characterized batches of PST material are shown.

## Data Availability

The raw data supporting the conclusions of this article will be made available by the authors on request.

## Appendix A. SEM Image and P-E-Loop

An SEM image of a PST sample is presented in Figure A1. The polarization *P* is plotted against the electric field strength *E* for various sample temperatures in a typical *P*-*E*-loop, as illustrated in Figure A2.

## Appendix B. Temperature Correction and Determination of the Temperature Uncertainty

### Temperature Correction

Figure A3 shows the temperature correction of the sample temperature. This procedure is used during the determination of the dissipative heat measurement to obtain a higher temperature accuracy.

## Appendix C. Separate Results for the Sample Batches

Figure A4 shows the separate measurement for the adiabatic temperature change and the dissipative heat measurement for both characterized batches. Figure A5 shows the result of the FOM calculation for both batches. All three graphs show the results plotted against the electric field strength.
